# Effects of Host Plant Factors on the Bacterial Communities Associated with Two Whitefly Sibling Species

**DOI:** 10.1371/journal.pone.0152183

**Published:** 2016-03-23

**Authors:** Ming-Ming Su, Lei Guo, Yun-Li Tao, You-Jun Zhang, Fang-Hao Wan, Dong Chu

**Affiliations:** 1 Key Lab of Integrated Crop Pest Management of Shandong Province, College of Agronomy and Plant Protection, Qingdao Agricultural University, Qingdao, 266109, P. R. China; 2 State Key Laboratory for Biology of Plant Diseases and Insect Pests, Institute of Plant Protection, Chinese Academy of Agricultural Sciences (CAAS), Beijing, 100081, P.R. China; 3 Department of Plant Protection, Institute of Vegetables and Flowers, Chinese Academy of Agricultural Sciences, Beijing, 100081, P. R. China; Volcani Center, ISRAEL

## Abstract

**Background:**

Although discrepancy in the specific traits and ecological characteristics of *Bemisia tabaci* between species are partially attributed to the *B*. *tabaci*-associated bacteria, the factors that affect the diversity of *B*. *tabaci*-associated bacteria are not well-understood. We used the metagenomic approach to characterize the *B*. *tabaci*-associated bacterial community because the approach is an effective tool to identify the bacteria.

**Methodology and Results:**

To investigate the effects of the host plant and a virus, *tomato yellow leaf curl virus* (TYLCV), on the bacterial communities of *B*. *tabaci* sibling species B and Q, we analyzed the bacterial communities associated with whitefly B and Q collected from healthy cotton, healthy tomato, and TYLCV-infected tomato. The analysis used miseq-based sequencing of a variable region of the bacterial 16S rDNA gene. For the bacteria associated with *B*. *tabaci*, we found that the influence of the host plant species was greater than that of the whitefly cryptic species. With further analysis of host plants infected with the TYLCV, the virus had no significant effects on the *B*. *tabaci*-associated bacterial community.

**Conclusions:**

The effects of different plant hosts and TYLCV-infection on the diversity of *B*. *tabaci*-associated bacterial communities were successfully analyzed in this study. To explain why *B*. *tabaci* sibling species with different host ranges differ in performance, the analysis of the bacterial community may be essential to the explanation.

## Introduction

The sweet potato whitefly, *Bemisia tabaci* (Gennadius) (Hemiptera: Aleyrodidae), is a complex of species that contains at least 36 morphologically indistinguishable species [1–4] that cause considerable damage to a wide range of agricultural, fiber, vegetable, and ornamental crops through both direct feeding and vectoring of geminiviruses such as *tomato yellow leaf curl virus* (TYLCV) [2, 5]. The specific traits and ecological attributes of the species in the complex are related to the *B*. *tabaci*-associated bacteria, in part. The symbioses between *B*. *tabaci* and endosymbionts are well-documented [6–8], but the factors that affect the diversity of other bacteria associated with *B*. *tabaci* are not well-understood.

The bacteria community associated with *B*. *tabaci* is a mix of mutualistic, pathogenic, and commensal bacteria. The primary symbionts compensate for the insufficient nutrients that *B*. *tabaci* obtains from a restricted diet of plant phloem [9]. In addition to providing nutrients, the secondary symbionts increase the susceptibility to insecticides [10, 11], improve the ability to transmit the TYLCV [12, 13], increase the thermotolerance [14], and increase the resistance to parasitoids [15]. Other bacteria are entomopathogenic and may act as biological control agents [16–18].

Based on recent metagenomic studies, the variation in gut-associated bacterial communities was dependent on the host plants in *Lymantria dispar* [19], *Helicoverpa armigera* [20, 21], *Drosophila melanogaster*, and *D*. *simulan* [22, 23], and on the diet in *Anopheles gambiae* [24]. The *B*. *tabaci* on different host plants have clearly different levels of performance [25, 26]. Additionally, the fitness and feeding behavior of *B*. *tabaci* were indirectly affected by the TYLCV obtained from the host tomato [27, 28]. Therefore, we hypothesized that the biotic factors of host plant and TYLCV-infection affected the diversity of the *B*. *tabaci-*associated bacteria.

However, until recently, all approaches to identify the *B*. *tabaci*-associated bacteria did not completely characterize the bacterial community, including isolation of the bacteria from *B*. *tabaci* that could be cultured [17, 18, 29, 30], amplification of bacterial 16S rDNA-specific primers [6–8, 31, 32], and a more thorough methodology of constructing a 16S rDNA clone library [33]. By contrast, metagenomic approaches provide a comprehensive characterization of bacterial community profiles, completely bypassing the use of cultures [34, 35].

To determine the effects of the biotic factors on the diversity of *B*. *tabaci*-associated bacteria, we analyzed the composition of the bacterial community of *B*. *tabaci* sibling species B (MEAM1 species, also known as biotype B) and Q (MED species, also known as biotype Q) on different host treatments (cultured on cotton, tomato, and TYLCV-infected tomato) with a metagenomic approach that used miseq-based sequencing of a variable region of the bacterial 16S rDNA gene.

## Materials and Methods

### Ethics statement

The research complied with all laws of the country (China) in which it was performed, and the research was approved by the Department of Science and Technology of the Qingdao Agricultural University, China (permit number: 20110712).

### *Bemisia tabaci* populations

The samples of *B*. *tabaci* sibling species B and Q used in this study were obtained from laboratory populations established from prior field collections. The details of the methods to maintain the populations are described in Fang et al. [36]. Briefly, the populations were maintained in separate climatic cubicles on cotton, *Gossypium hirsutum* (Malvaceae) cv. Lu-Mian 21, a host plant suitable to both *B*. *tabaci* B and Q. Using the *Vsp* I-based *mtCOI* PCR-RFLP method [37, 38], the purity of each population was monitored every 30 days with a sample of 20 adults.

Two species of crop plants were used in this study: (i) cotton, cv. Lu-Mian 21, and (ii) tomato, *Lycopersicon esculentum* (Solanaceae) cv. Zhe-Fen 212, which included healthy plants and those infected with the TYLCV. Three types of treatments were used in the study. In the first type of treatment, the populations were maintained in separate climatic cubicles on cotton, a host plant suitable to both *B*. *tabaci* B and Q (the BC and QC groups, respectively), for one generation. In the second type of treatment, the populations of B and Q were transferred to healthy tomato plants from cotton (the BT and QT groups, respectively) and were maintained for one generation. In the third type of treatment, the populations of B and Q were transferred from cotton to tomato infected with the TYLCV (the BTV and QTV groups, respectively) and were maintained for one generation. All experiments used plants at the 5–7 fully expanded true leaf stage and were conducted in climate chambers (27 ± 1°C, 16L:8D, and 60 ± 5% RH). All samples were collected in the 2nd generation and stored at -20°C.

### DNA extraction and sequencing

Each insect sample (comprising 20 adult female whiteflies) and the cotton leaf sample were rinsed at least three times in 75% ethanol. The insect and cotton leaf genomic DNA were extracted from the samples using the TIANamp Genomic DNA kit and Plant Genomic DNA kit (TIANGEN Biotech Co., Ltd, Beijing, China), respectively.

Amplicon liberates were constructed for miseq-sequencing using bacterial fused primers 341F (5’-CCTACACGACGCTCTTCCGATCTN-barcode-CCTACGGGNGGCWGCAG-3’) and 805R (5’-GACTGGAGTTCCTTGGCACCCGAGAATTCCA-barcode- GACTACHVGGGTATCTAATCC-3’) for the V3-V4 region of the 16S rDNA [39]. The barcode fragments were used to sort multiple samples in a single sequencing run. PCR reactions were performed in 50 ul buffer containing 1×PCR buffer, 1 mM dNTPs, 5 uM each primer, 1 U Plantium Taq and 10 ng of template DNA. The PCR was performed under the following conditions: 94°C for 3 min, followed by 5 cycles of 94°C for 30 sec, 45°C for 20 sec, and 65°C for 30 sec, then followed by 20 cycles of 94°C for 20 sec, 55°C for 20 sec, 72°C for 30 sec, and 72°C for 5 min.

The products of the amplicon of the 16S rDNA from different samples were pooled in equimolar ratio, and then added the library barcodes on the Illumina PE adapters to construct the PCR amplicon libraries, and finally carried out on an Illumina Miseq for sequencing. The raw data have been deposited in the Sequence Read Archive (SRA) database under accession number SRS1022467.

### Statistical analyses

The sequences were grouped into OTUs using uclust software (uclust v1.1.579) with the 97% identity thresholds. The richness rarefaction curves, Shannon index, ACE, Chao1, and coverage were calculated with Mothur analyses [40]. The RDP classifier was used to assign sequences to phylogenetic taxonomy based on Bergey’s taxonomy using Ribosomal Database Project [41, 42], and the sequences were assigned to the hierarchical taxa under the condition of bootstrap cutoff at 80%. The number of genera was analyzed using one-way ANOVA in Sigmaplot v.12.0 software. The stem-and-leaf figure with the most abundant genera of bacteria associated with *B*. *tabaci* was constructed with SPSS v.19.0 software. The statistical significance of differences in abundance in the bacterial community associated with *B*. *tabaci* among treatments was determined with student *t*-tests. The principal coordinates analysis (PCoA) was conducted using the unifrac metric [39].

## Results

### Overview of *B*. *tabaci*-associated bacterial community

**Fig 1:**
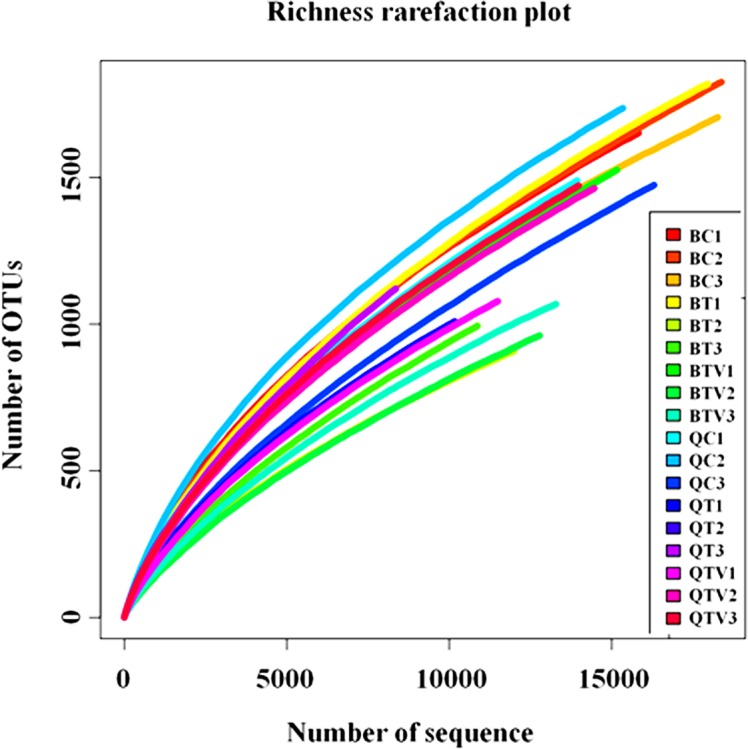
Rarefaction analysis of the different samples. Rarefaction curves of OTUs (operational taxonomic units) clustered at 97% sequence identity for different samples.

To evaluate the diversity and richness of the *B*. *tabaci*-associated bacterial community, rarefaction curves, Good’s coverage, ACE, Chao1, and Shannon parameters were applied to estimate these qualities (Fig 1 and S1 Table). The rarefaction curves (Fig 1) were generated by plotting the number of phylotypes (operational taxonomic units, OTUs) against the number of identified sequences. None of the rarefaction curves of treatment samples reached a plateau, which indicated that even with over 10000 sequences sampled for each treatment sample, the number of OTUs was likely to increase with additional sampling.

**Fig 2:**
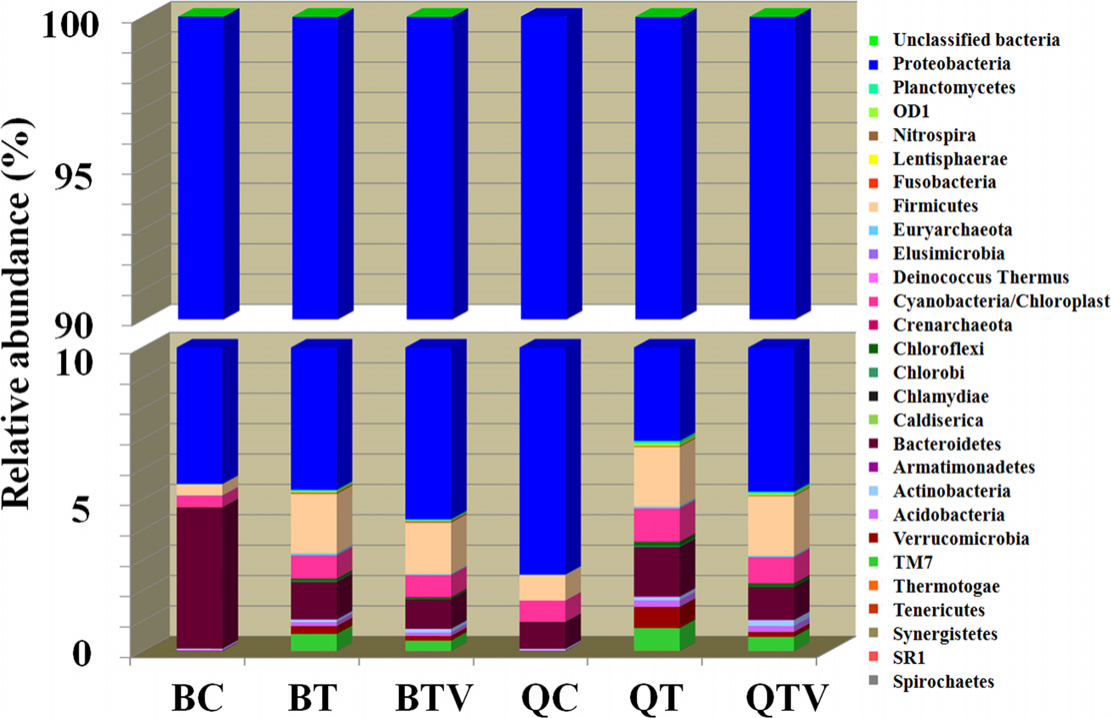
The relative abundance of bacterial phyla in each group. Sequences that could not be classified into any known group were assigned as ‘Unclassified bacteria’.

For the overall bacterial community associated with *B*. *tabaci*, 27 different phyla were identified (Fig 2). However, *Proteobacteria* was the most important group in all samples, representing above 90.00% of the community (Fig 2).

### Bacterial community associated with *B*. *tabaci* B and Q on cotton

**Fig 3:**
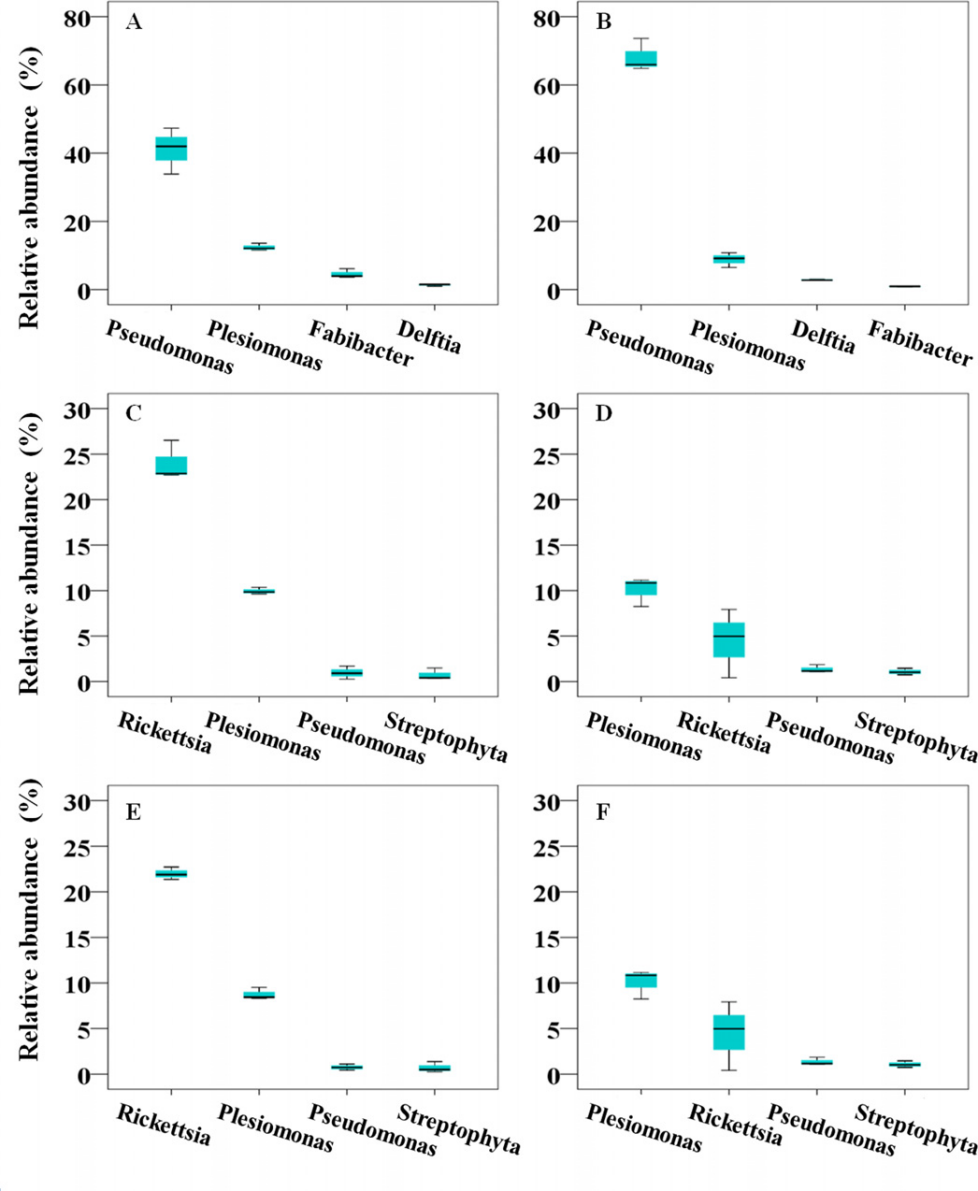
The most abundant bacterial genera associated with *B*. *tabaci*. (A) BC group, (B) QC group, (C) BT group, (D) QT group, (E) BTV group, and (F) QTV group. Plotted values are mean relative abundance of the genus.

For the overall bacterial community associated with *B*. *tabaci* on cotton, 15 phyla were identified from both *B*. *tabaci* B and Q (Fig 2). With a small shift in range, 132 and 144 genera were associated with *B*. *tabaci* B and Q, respectively. The most prevalent genera with extreme ranges in the community associated with BC were the following: *Pseudomonas* (41.08%, range: 33.89–47.35%), *Plesiomonas* (12.42%, range: 11.59–13.59%), *Fabibacter* (4.59%, range: 3.68–6.14%), and *Delftia* (1.35%, range: 1.00–1.54%) (Fig 3A). The predominant genera in the community associated with QC were the following: *Pseudomonas* (68.16%, range: 64.88–73.64%), *Plesiomonas* (8.81%, range: 6.48–10.80%), *Delftia* (2.83%, range: 2.68–3.04%), and *Enterobacter* (0.90%, range: 0.80–0.96%) (Fig 3B).

### Bacterial community associated with *B*. *tabaci* B and Q on healthy tomato

For the overall bacterial community associated with *B*. *tabaci*, 22 phyla were identified from both *B*. *tabaci* B and Q from healthy tomato (Fig 2). With a small shift in range, 397 and 329 genera were associated with *B*. *tabaci* B and Q, respectively. The most prevalent genera with extreme ranges in the community associated with BT were the following: *Rickettsia* (24.03%, range: 22.70–26.52%), *Plesiomonas* (9.95%, range: 9.64–10.36%), *Pseudomonas* (0.96%, range: 0.28–1.70%), and *Streptophyta* (0.74%, range: 0.34–1.48%) (Fig 3C). The predominant genera in the community associated with QT were *Plesiomonas* (10.08%, range: 8.24–11.16%), *Rickettsia* (4.44%, range: 0.41–7.93%), *Pseudomonas* (1.35%, range: 1.06–1.85%), and *Streptophyta* (1.07%, range: 0.74–1.46%) (Fig 3D).

### Bacterial community diversity associated with *B*. *tabaci* B and Q on TYLCV-infected tomato

For the overall bacterial community associated with *B*. *tabaci*, 21 and 23 phyla were identified associated with *B*. *tabaci* B and Q from TYLCV-infected tomatoes, respectively (Fig 2). With a small shift in range, 388 and 395 genera were associated with *B*. *tabaci* B and Q, respectively. The most prevalent genera with extreme ranges in the community associated with BTV were the following: *Rickettsia* (22.00%, range: 21.37–22.72%), *Plesiomonas* (8.76%, range: 8.33–9.52%), *Pseudomonas* (0.76%, range: 0.44–1.11%), and *Streptophyta* (0.72%, range: 0.27–1.38%). The predominant genera in the community associated with QTV were *Plesiomonas* (10.95%, range: 9.87–12.66%), *Rickettsia* (6.91%, range: 0.04–3.62%), *Pseudomonas* (0.93%, range: 0.82–1.07%), *Streptophyta* (0.84%, range: 0.75–1.01%) (Fig 3C).

### Difference in *B*. *tabaci*-associated bacterial diversity between cotton and tomato populations

**Fig 4:**
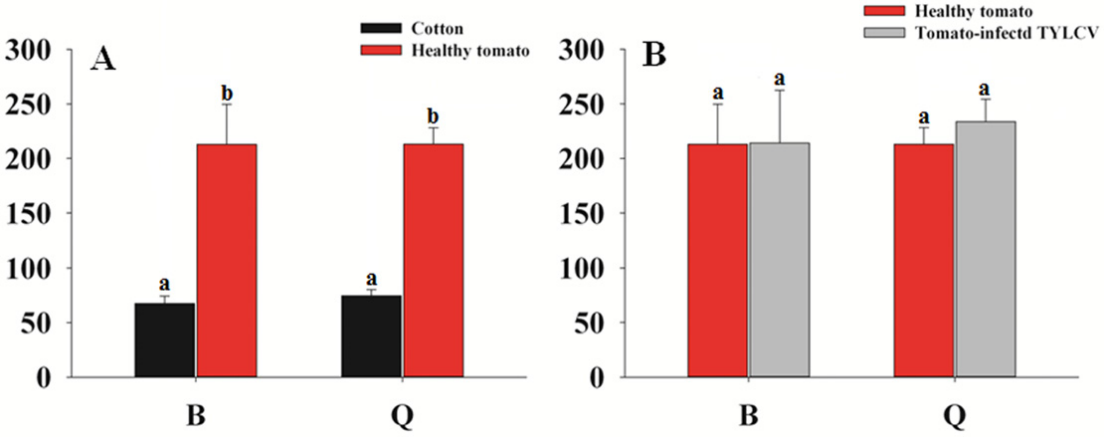
Comparison of the mean number of genera of *B*. *tabaci*-associated bacteria from *B*. *tabaci* raised in different plants. (A) Comparison between that in cotton and healthy tomato (B) Comparison between that in healthy tomato and tomato-infected TYLCV.

The difference in *B*. *tabaci*-associated bacterial diversity between cotton and tomato populations was obvious (Fig 4A). The number of genera between QC and QT was significantly different, and the tendency was the same between BC and BT (Fig 4A). Additionally, the abundance of 68 genera was significantly different between BC and BT groups, and the abundance of 55 genera was clearly different between QC and QT groups (S2 Table).

**Fig 5:**
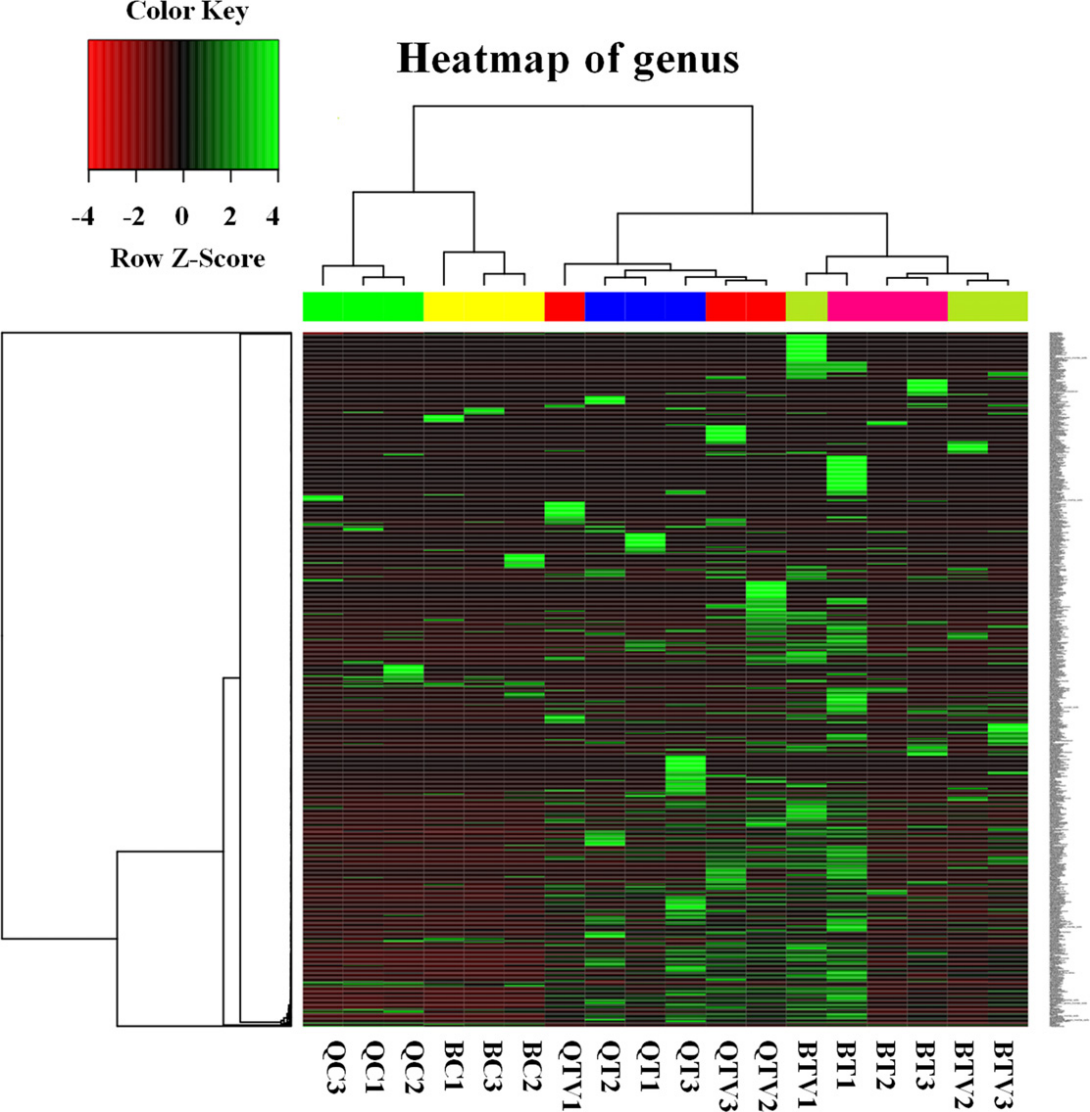
Heatmap of relative abundances of the main genera associated with *B*. *tabaci* from each group based on 16S rDNA sequences. Complete linkage clustering of 18 samples based on genera composition and relative abundance in communities. Each row is an individual genus, and each column is a sample. Color key and color bars are in the top-left corner.

A correlogram of the bacterial community associated with *B*. *tabaci* was analyzed and was presented using heatmaps at the level of genus (Fig 5). The heatmaps showed the BC and QC samples grouped together, and others grouped together as well. The PCoA analyses based on the weighted unifrac distance metric [43] were conducted, and the bacterial communities associated with *B*. *tabaci* B and Q fed on cotton had little variance between them (S1 Fig).

### Difference in *B*. *tabaci*-associated bacterial diversity between healthy and TYLCV-infected tomato populations

The *B*. *tabaci*-associated bacterial diversity was not significantly different between healthy and TYLCV-infected tomato populations. The number of genera between QC and QT was significantly different, and the tendency was the same between BC and BT (Fig 4B). The abundance of nine genera was significantly different between BT and BTV groups, and the abundance of six genera clearly differed between QT and QTV groups (S3 Table).

A correlogram of the bacterial community associated with *B*. *tabaci* was analyzed and presented using heat maps at the level of genus (Fig 5). The heatmaps showed the BT and BTV samples grouped together, and the QT and QTV grouped together as well. The PCoA analysis found that the bacterial communities associated with *B*. *tabaci* B fed healthy tomato had little variance, compared to TYLCV-infected tomato, and the same as in *B*. *tabaci* Q (S1 Fig).

### Similarity between the bacterial genera in cotton leaf and that in *Bemisia tabaci* Q

The similarity analysis between the bacterial genera in cotton leaf and that in *B*. *tabaci* Q was analyzed, and the result showed that there were 50.91%, 62.25%, and 53.39% genera in three host whitefly (QC) can be found in cotton leaf, respectively.

## Discussion

### Bacterial community associated with *B*. *tabaci* B and Q

Our protocol of using a metagenomic approach that used miseq-based sequencing of a variable region of the bacterial 16S rDNA gene provide the complete picture of bacteria associated with *B*. *tabac*i, more than 300 genera including unculturable symbionts, culturable bacteria and unculturable bacteria, showing that many more bacteria are associated with *B*. *tabaci* than previously described [33].

Morever, the predominant phylum of the communities in the six groups was *Proteobacteria*, which composed over 90% of the community. Oesi-Poku et al. [44] found that *Proteobacteria* was typically the predominant bacterial taxon in the gut of mosquitoes, which was consistent with the reports of Wang et al. [24] and Jones et al. [45].

### Factors affecting *B*. *tabaci*-associated bacterial diversity

This study showed that the host plant played an important role in shaping the composition of the bacterial community associated with *B*. *tabaci*. Our results can also be supported by Pan et al. [46] that host plant can affect the relative amount of symbionts such as *Portiera*, *Cardinium*, *Rickettsia*, and *Hamiltonella* in *B*. *tabaci*. We have further analyzed the similarity between the bacterial genera in cotton leaf and those in *B*. *tabaci* Q, which confirmed the important role that the host plant played in shaping the composition of the bacterial community in insects.

Our result also revealed that the host plant played a more important role in shaping the composition of the bacterial community associated with *B*. *tabaci* than the cryptic species. This result was consistent with Chandler et al. [22] that host diet has a greater effect on the bacterial microbiome composition in *Drosophila*, than *Drosophila* species. Anderson et al. [47] also found that highly similar bacterial communities were shared among related and trophically similar herbivorous ant species.

These results can be explained by two possibilities. One possibility was that the gut-associated bacteria have relatively high ratio in the bacterial community associated with *B*. *tabaci* while the gut-associated bacteria were mainly obtained from the host plant [17]. For example, many bacterial taxa frequently reported in plants are the genera *Pseudomonas*, *Bradyrhizobium*, *Azorhizobium*, *Azospirillum*, and *Bacillus* [48]. Of these genera, *Pseudomonas* and *Bacillus* were detected in *B*. *tabaci* in this study, which may be obtained from host plant. Alternatively, the same plant could provide a suitable living environment for the same bacteria in the guts in different whitefly species, and thus different cryptic species of *B*. *tabaci* feeding on the same host plant may have high similarity of bacterial composition.

The TYLCV had almost no effect on the bacterial community associated with *B*. *tabaci*. In previous work that compared the feeding behaviors of *B*. *tabaci* B and Q on TYLCV-infected tomatoes, the B and Q also responded similarly to infected plants, and no differences were found [27], which indicated that the TYLCV did not change the plant-associated bacterial community. However, the normal route of bacterial invasion is via oral ingestion [49, 50], which might explain why the TYLCV had no effect on the *B*. *tabaci*-associated bacterial community.

In this study, *Hamiltonella* was not detected in *B*. *tabaci* B, which is inconsistent with the previous studies [51–53] that the infection frequencies of *Hamiltonella* collected from field populations of *B*. *tabaci* B ranged from 46.70% to 100%. 454 pyrosequencing of 16S rRNA gene sequences showed that the relative abundance of *Hamiltonella* range from 1% to 50% in seven field populations of *B*. *tabaci* B from Israel [54]. Two possibilities might explain the discrepancy. One possibility is that different primers may result in different abundance of a certain bacteria. We speculate the primers used in this study might not yield the amplicons of the 16S rRNA of *Hamiltonella* in whitefly. Another possibility is that the different databases were used to identify the bacterium. Ribosomal Database Project was used to assign sequences to phylogenetic taxonomy in this study, while the NCBI StandAlone BLAST (megablast program) was used to identity bacterial species in Jing et al. [54].

### Future Research

In this study, we found that host plants had significant effects on the relative amounts of *B*. *tabaci*-associated bacteria, such as *Rickettsia*. This result was consistent with Pan et al. [46, 55], who reported a significant change in the abundance of symbionts among different host plant-adapted *B*. *tabaci* B and Q. And the *Rickettsia* in *B*. *tabaci* had some involvement with the resistance against insecticides [10, 11]. However, the whitefly cryptic species that were maintained on different host plants had different susceptibilities to insecticides [56–58]. Therefore, a hypothesis is proposed that host plants influence the *B*. *tabaci*-associated bacteria, which thereby affect the performance of *B*. *tabaci*, for example, in the susceptibility to insecticides. It requires further research.

## Supporting Information

S1 Fig Principal coordinates analysis (PCoA) of weighted unifrac distances of 16S rDNA. Scatter plot of PCA scores depicting variance of fingerprints derived from different *B*. *tabaci*-associated bacterial communities.
(TIF)

S1 Table Sequencing data with richness and diversity estimation of bacterial taxa in six groups of *Bemisia tabaci*. (DOC)

S2 Table The genus-level comparison of bacterial composition associated with *B*. *tabaci* from healthy tomato and cotton. (DOC)

S3 Table The genus-level comparison of bacterial composition associated with *B*. *tabaci* from healthy tomato plants and TYLCV- infected tomato. (DOC)
