# Supplementary figures and images for: Effects of Host Plant Factors on the Bacterial Communities Associated with Two Whitefly Sibling Species
Source: PLoS One. 2016 Mar 23;11(3):e0152183. doi: 10.1371/journal.pone.0152183 (PMC4805303; doi:10.1371/journal.pone.0152183)

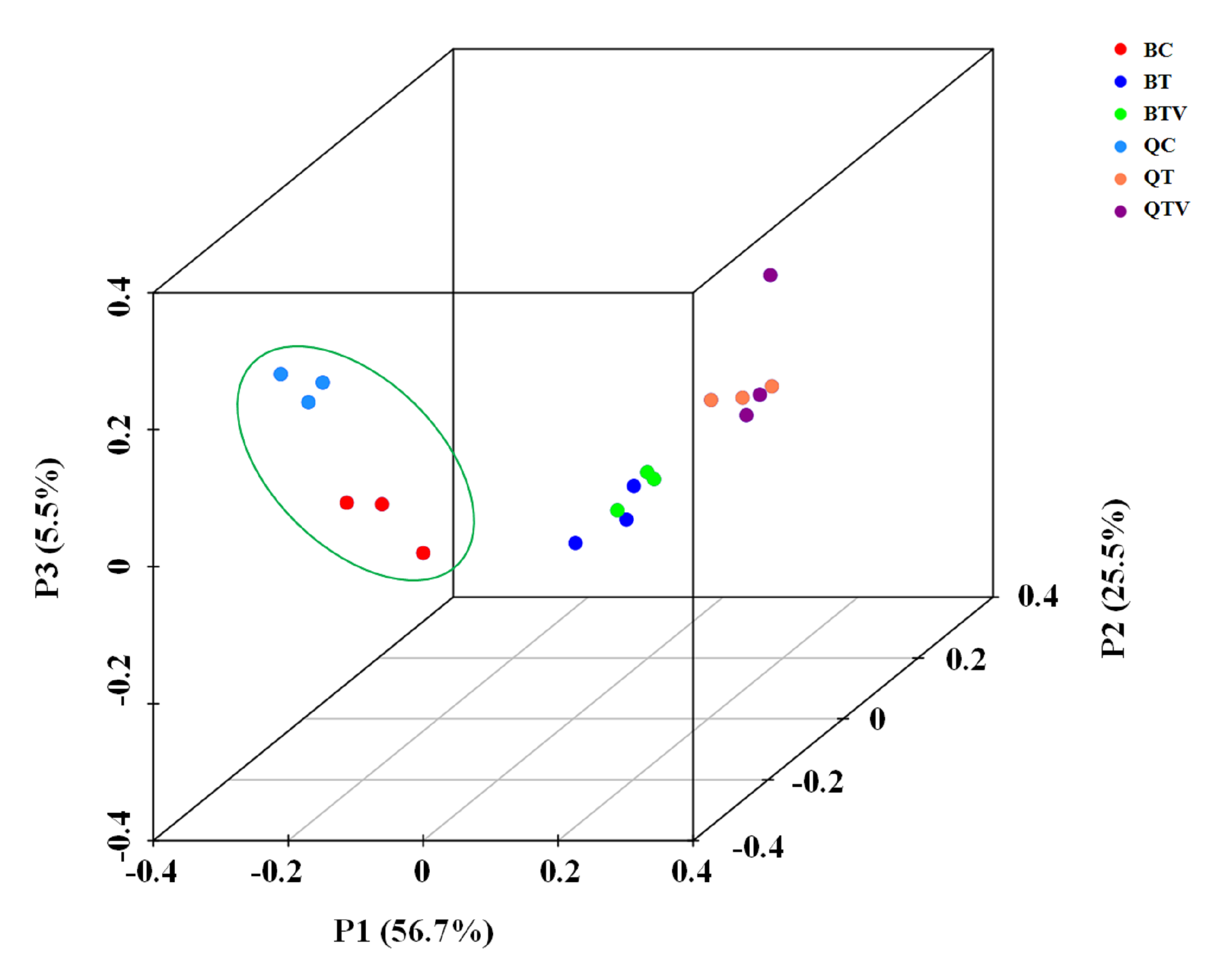

Supplement: S1 Fig — Scatter plot of PCA scores depicting variance of fingerprints derived from different B. tabaci-associated bacterial communities. (TIF) [file pone.0152183.s001.tif]
