# Supplementary material for: Effects of Host Plant Factors on the Bacterial Communities Associated with Two Whitefly Sibling Species
Source: PLoS One. 2016 Mar 23;11(3):e0152183. doi: 10.1371/journal.pone.0152183 (PMC4805303; doi:10.1371/journal.pone.0152183)
Supplement: S1 Table — (DOC) [file pone.0152183.s002.doc]

**Table S1. Sequencing data with richness and diversity estimation of bacterial taxa in six groups of *Bemisia tabaci*.**

| **Sample** | **Cut off** | **Reads** | **OTUs** | **Shannon_index** | **ACE_index** | **Chao1_index** | **Coverage** |
| --- | --- | --- | --- | --- | --- | --- | --- |
| BC1 | 0.03 | 15823 | 1034 | 2.97 | 3832.71 | 2390.44 | 0.96 |
| BC2 | 0.03 | 18380 | 1224 | 3.16 | 5302.53 | 3501.42 | 0.96 |
| BC3 | 0.03 | 18268 | 1117 | 3.18 | 4597.02 | 3051.29 | 0.96 |
| QC1 | 0.03 | 13937 | 1116 | 3.54 | 4416.02 | 2983.26 | 0.95 |
| QC2 | 0.03 | 15346 | 1260 | 3.62 | 4354.62 | 2803.28 | 0.95 |
| QC3 | 0.03 | 16305 | 1175 | 3.16 | 5313.79 | 3465.41 | 0.95 |
| BT1 | 0.03 | 17957 | 1528 | 3.43 | 4287.88 | 3035.63 | 0.95 |
| BT2 | 0.03 | 11995 | 686 | 2.37 | 2701.62 | 1748.53 | 0.96 |
| BT3 | 0.03 | 10869 | 775 | 2.54 | 3193.28 | 1963.10 | 0.95 |
| BTV1 | 0.03 | 15167 | 1277 | 3.25 | 4094.31 | 2649.74 | 0.95 |
| BTV2 | 0.03 | 12784 | 733 | 2.43 | 3559.49 | 2077.92 | 0.96 |
| BTV3 | 0.03 | 13285 | 867 | 2.60 | 2995.79 | 1917.02 | 0.96 |
| QT1 | 0.03 | 10171 | 789 | 2.67 | 3151.27 | 1925.34 | 0.95 |
| QT2 | 0.03 | 7148 | 822 | 2.55 | 4153.72 | 2079.09 | 0.92 |
| QT3 | 0.03 | 8359 | 946 | 3.08 | 3822.11 | 2400.88 | 0.93 |
| QTV1 | 0.03 | 11495 | 838 | 2.29 | 2879.60 | 1994.50 | 0.96 |
| QTV2 | 0.03 | 14465 | 1186 | 2.99 | 4234.90 | 2652.44 | 0.95 |
| QTV3 | 0.03 | 13980 | 1152 | 3.21 | 3663.26 | 2370.68 | 0.95 |

The operational taxonomic units (OTUs) were defined at 3% dissimilarity level. The coverage percentage (Good), richness estimators (ACE and Chao1) and diversity indices (Shannon) were calculated by the Mothur analysis.
