# Supplementary material for: Effects of Host Plant Factors on the Bacterial Communities Associated with Two Whitefly Sibling Species
Source: PLoS One. 2016 Mar 23;11(3):e0152183. doi: 10.1371/journal.pone.0152183 (PMC4805303; doi:10.1371/journal.pone.0152183)
Supplement: S2 Table — (DOC) [file pone.0152183.s003.doc]

**Table S2. The genus-level comparison of bacterial composition associated with *B. tabaci* from healthy tomato and cotton.**

| ***B. tabac*i-associated bacteria** | **B** | **Q** |
| --- | --- | --- |
|  | ***P* value** | ***P* value** |
| *Acetobacter* | **＊＊＊** | **＊＊** |
| *Acidovorax* | **＊＊** |  |
| *Acinetobacter* | **＊＊＊** | **＊＊＊** |
| *Aeromonas* | **＊＊＊** | **＊** |
| *Armatimonadetes_gp5* | **＊** | **＊** |
| *Azomonas* |  | **＊＊** |
| *Azorhizophilus* | **＊＊＊** | **＊＊＊** |
| *Azotobacter* | **＊＊＊** | **＊＊** |
| *Bacillus* | **＊＊＊** | **＊＊＊** |
| *Bacteroides* |  | **＊** |
| *Bellilinea* |  | **＊＊** |
| *Brevundimonas* | **＊** |  |
| *Brucella* | **＊＊＊** | **＊＊** |
| *Buttiauxella* | **＊** |  |
| *Castellaniella* | **＊** |  |
| *Catellibacterium* | **＊＊** | **＊** |
| *Clostridium IV* | **＊＊** | **＊** |
| *Clostridium sensu stricto* | **＊＊** |  |
| *Comamonas* | **＊** |  |
| *Delftia* | **＊＊＊** | **＊＊＊** |
| *Devosia* | **＊＊＊** | **＊＊＊** |
| *Dokdonella* | **＊** |  |
| *Ensifer* | **＊** |  |
| *Enterobacter* | **＊＊＊** | **＊＊＊** |
| *Enterococcus* | **＊＊＊** | **＊** |
| *Erythrobacter* | **＊** |  |
| *Escherichia/Shigella* |  | **＊＊＊** |
| *Fabibacter* | **＊＊＊** | **＊＊＊** |
| *Ferruginibacter* | **＊＊** |  |
| *Flavobacterium* | **＊＊＊** | **＊＊＊** |
| *Fulvivirga* | **＊** |  |
| *Gillisia* | **＊＊＊** | **＊＊＊** |
| *Gp4* | **＊＊** | **＊＊＊** |
| *Haliscomenobacter* | **＊** | **＊＊** |
| *Halotalea* | **＊＊** |  |
| *Ignavibacterium* | **＊** | **＊** |
| *Lactobacillus* | **＊＊＊** | **＊＊＊** |
| *Lactococcus* | **＊＊** | **＊＊** |
| *Levilinea* | **＊** | **＊** |
| *Lutibacter* |  | **＊** |
| *Lysobacter* | **＊** | **＊** |
| *Massilia* | **＊＊＊** | **＊＊＊** |
| *Mesorhizobium* |  | **＊** |
| *Methylibium* | **＊** | **＊＊** |
| *Niastella* |  | **＊** |
| *Nitriliruptor* | **＊** |  |
| *Novosphingobium* | **＊＊＊** | **＊＊** |
| *OD1_genera_incertae_sedis* | **＊＊＊** | **＊＊＊** |
| *Ohtaekwangia* | **＊＊＊** | **＊** |
| *Plesiomonas* | **＊＊＊** | **＊＊＊** |
| *Prolixibacter* | **＊** | **＊** |
| *Propionivibrio* | **＊** | **＊** |
| *Pseudomonas* | **＊＊＊** | **＊＊＊** |
| *Psychrobacter* | **＊** |  |
| *Rhizobium* | **＊＊＊** |  |
| *Rhodobacter* | **＊** |  |
| *Rickettsia* | **＊** | **＊＊＊** |
| *Shewanella* | **＊＊＊** | **＊＊＊** |
| *Simplicispira* | **＊＊＊** | **＊** |
| *Spartobacteria_genera_incertae_sedis* | **＊＊＊** | **＊＊＊** |
| *Sphingomonas* | **＊＊＊** | **＊＊＊** |
| *Sphingopyxis* | **＊＊＊** | **＊** |
| *Stenotrophomonas* | **＊＊＊** | **＊＊＊** |
| *Streptococcus* | **＊＊＊** |  |
| *Streptophyta* | **＊＊＊** | **＊＊＊** |
| *Subdivision3_genera_incertae_sedis* |  | **＊＊＊** |
| *Terribacillus* | **＊＊＊** | **＊＊＊** |
| *Terrimonas* | **＊＊＊** | **＊** |
| *Thauera* | **＊** |  |
| *Thiobacillus* | **＊** | **＊** |
| *Thiohalophilus* | **＊＊＊** |  |
| *Thioprofundum* | **＊＊＊** | **＊＊＊** |
| *TM7_genera_incertae_sedis* | **＊＊＊** | **＊＊＊** |
| *Verrucomicrobium* | **＊** | **＊** |
| *Weissella* | **＊** |  |

*P* value were analyzed from Student t-test, and **^＊^**represent ＜0.05, **^＊＊^**represent ＜0.01, **^＊＊＊^**represent ＜0.001.
