# Supplementary material for: Effects of Host Plant Factors on the Bacterial Communities Associated with Two Whitefly Sibling Species
Source: PLoS One. 2016 Mar 23;11(3):e0152183. doi: 10.1371/journal.pone.0152183 (PMC4805303; doi:10.1371/journal.pone.0152183)
Supplement: S3 Table — (DOC) [file pone.0152183.s004.doc]

**Table S3. The genus-level comparison of bacterial composition associated with *B. tabaci* from healthy tomato plants and TYLCV- infected tomato.**

| ***B. tabaci*-associated bacteria** | **B** | **Q** |
| --- | --- | --- |
|  | ***P* value** | ***P* value** |
| *Devosia* | **＊＊** |  |
| *Enterobacter* |  | **＊＊＊** |
| *Escherichia/Shigella* | **＊＊** | **＊＊＊** |
| *Ohtaekwangia* | **＊** |  |
| *Plesiomonas* | **＊＊** |  |
| *Pseudomonas* | **＊** | **＊＊** |
| *Rickettsia* | **＊＊＊** | **＊＊＊** |
| *Spartobacteria_genera_incertae_sedis* | **＊＊** | **＊＊＊** |
| *TM7_genera_incertae_sedis* | **＊＊＊** | **＊** |

*P* value were analyzed from Student t-test, and **^＊^**represent ＜0.05, **^＊＊^**represent ＜0.01, **^＊＊＊^**represent ＜0.001.
